# Adrenomedullin Inhibits Osmotic Water Permeability in Rat Inner Medullary Collecting Ducts

**DOI:** 10.3390/cells9122533

**Published:** 2020-11-24

**Authors:** Fuying Ma, Guangping Chen, Eva L. Rodriguez, Janet D. Klein, Jeff M. Sands, Yanhua Wang

**Affiliations:** 1 Renal Division, Department of Medicine, Emory University, Atlanta, GA 30322, USA; fuying.ma@emory.edu (F.M.); eva.rodriguez@emory.edu (E.L.R.); janet.klein@emory.edu (J.D.K.); jeff.sands@emory.edu (J.M.S.); 2 Department of Physiology, Emory University, Atlanta, GA 30322, USA; gchen3@emory.edu

**Keywords:** adrenomedullin, water transport, cAMP, phospholipase C, protein kinase C, cGMP

## Abstract

Adrenomedullin (ADM) is a vasodilator that causes natriuresis and diuresis. However, the direct effect of ADM on osmotic water permeability in the rat inner medullary collecting duct (IMCD) has not been tested. We investigated whether ADM and its ADM receptor components (CRLR, RAMP2, and 3) are expressed in rat inner medulla (IM) and whether ADM regulates osmotic water permeability in isolated perfused rat IMCDs. The mRNAs of ADM, CRLR, and RAMP2 and 3 were detected in rat IM. Abundant protein of CRLR and RAMP3 were also seen but RAMP2 protein level was extremely low. Adding ADM (100 nM) to the bath significantly decreased osmotic water permeability. ADM significantly decreased aquaporin-2 (AQP2) phosphorylation at Serine 256 (pS256) and increased it at Serine 261 (pS261). ADM significantly increased cAMP levels in IM. However, inhibition of cAMP by SQ22536 further decreased ADM-attenuated osmotic water permeability. Stimulation of cAMP by roflumilast increased ADM-attenuated osmotic water permeability. Previous studies show that ADM also stimulates phospholipase C (PLC) pathways including protein kinase C (PKC) and cGMP. We tested whether PLC pathways regulate ADM-attenuated osmotic water permeability. Blockade of either PLC by U73122 or PKC by rottlerin significantly augmented the ADM-attenuated osmotic water permeability and promoted pS256-AQP2 but did change pS261-AQP2. Inhibition of cGMP by L-NAME did not change AQP2 phosphorylation. In conclusion, ADM primarily binds to the CRLR-RAMP3 receptor to initiate signaling pathways in the IM. ADM reduced water reabsorption through a PLC-pathway involving PKC. ADM-attenuated water reabsorption may be related to decreased trafficking of AQP2 to the plasma membrane. cAMP is not involved in ADM-attenuated osmotic water permeability.

## 1. Introduction

Adrenomedullin (ADM) is a vasodilator peptide first extracted from human pheochromocytoma tissue in 1993 [1]. It is a 52-amino acid peptide that belongs to the calcitonin/calcitonin gene-related peptide (CGRP) family, which includes calcitonin, CGRP, and amylin. ADM receptors are type II G protein-coupled receptors [2], and each ADM receptor is formed by a complex of calcitonin-receptor-like receptor (CRLR) and receptor-activity-modifying protein 2 or 3 (RAMP2 or RAMP3). RAMPs conjugate with CRLR in the endoplasmic reticulum and facilitate the glycosylation and transport of CRLR to the cell surface [3,4]. ADM binds to the receptor of CRLR-RAMP 2 or 3, resulting in stimulation of adenylyl cyclase and phospholipase C (PLC) [5]. Subsequently, adenylyl cyclase activates cyclic adenosine monophosphate (cAMP) signaling pathways [1,2,6] and PLC activates cyclic guanosine monophosphate (cGMP) and protein kinase C (PKC) pathways [5]. Since it was identified, ADM has been shown to be expressed in various tissues including adrenal medulla, vascular endothelial and smooth muscle cells, central nervous system, pituitary gland, kidney, and cultured mesangial cells [6,7,8,9]. The kidney is thought to be a major producer of ADM since renal tissue and urinary levels (12 ± 1.5 fmol/mL) of ADM are much higher than plasma levels (2.1 ± 0.7 fmol/mL) [8,10]. In the kidney, the mRNA for ADM is most abundant in the cortex but is also found in the medullary collecting ducts [9,11]. The mRNA for ADM receptors is detected in the cortex but is most abundant in the inner medulla (IM) [11]. The inconsistent distribution of ADM and its receptors suggest that ADM may be produced in the cortex but is used in the IM.

In addition to the vasodilating effects, ADM has actions on fluid and electrolyte homeostasis [5,12]. ADM causes natriuresis and diuresis, which is associated with increases in glomerular filtration rate and a decrease in distal tubular sodium reabsorption [13,14]. In the brain, ADM inhibits water reabsorption and salt appetite [5]. In contrast, data from Leclerc et al. show that ADM increases sodium reabsorption in rabbit distal tubules [15], which suggests a subsequent increase in water reabsorption. These paradoxical findings suggest that further investigation needs to be performed to clarify the effect of ADM on fluid and electrolyte homeostasis in renal tubules.

In the kidney, the IM is the major site where vasopressin (AVP) and other hormones regulate water reabsorption [16,17,18]. Aquaporin 2 (AQP2) is the primary water channel that is expressed in the IMCD and stimulated by vasopressin [19,20]. AQP2, located at the apical plasma membrane and the subapical vesicles in collecting duct principal cells, is regulated by vasopressin-stimulated increases in both its phosphorylation and its apical plasma membrane accumulation [21]. Given that the mRNA for ADM receptors is abundant in the IM, the IM may also be the site where ADM exerts its effects on water reabsorption.

In this study, we investigated whether ADM affects osmotic water permeability in rat IMCDs. Since ADM can activate cAMP, PLC, cGMP, and PKC, we also investigated whether these signaling molecules are involved in the regulation of ADM-mediated osmotic water permeability. This is the first study that identifies the expression of ADM receptors at the protein level and the signaling pathways through which ADM regulates osmotic water permeability in renal IM.

## 2. Materials and Methods

### 2.1. Animals

All animal surgical protocols and procedures were approved by the Emory Institutional Animal Care and Use Committee (protocol number “PROTO 201800110”, approved on 21 December 2017, renewed on 2 September 2020, expires on 1 September 2023) and adhere to NIH standards for animal use. These studies used both male and female rats from Charles River Laboratories, Wilmington, MA, USA. To measure osmotic water permeability (Pf), rats weighing 50–75 g were sacrificed by decapitation to avoid any anesthesia effect and the kidneys quickly dissected to remove the inner medullas. The rats used in this study are 3–4 weeks old. Urine concentrating ability is fully developed at that age and the tubules are in good condition for dissection. To test AQP2 phosphorylation, rats weighing 100–140 g were sacrificed, and the IMs placed on ice until tissue treatments.

### 2.2. Tubule Perfusion

IMs were transferred to a dissection dish and the terminal IMCDs microdissected in a dissection buffer at 17 °C. The dissecting solution contained (in mM): 125 NaCl, 25 NaHCO_3_, 2 CaCl_2_, 2.5 K_2_HPO_4_, 1.2 MgSO_4_, 5.5 glucose. The dissecting solution had a pH of 7.4 and an osmolality of 430 mosmol/kg H_2_O [22]. The perfusion and bath solutions were identical to the dissection medium in chemical composition, except that 5 mM raffinose was added to both the perfusate and the bath and an additional 70 mM NaCl was added to the bath when measuring osmotic water permeability to create a bath-to-lumen osmolality gradient of ∼140 mosmol/kg H_2_O. Both the perfusion and bath solutions had a pH of 7.4. The perfusion solution had an osmolality of 290 mosmol/kg H_2_O. The bath solution had an osmolality of 430 mosmol/kg H_2_O. All solutions were gassed continuously with 95% air and 5% CO_2_ before and during the dissection and perfusion.

Single IMCDs were dissected, mounted on glass pipettes, and perfused as described [16]. In general, 45 min after warming the tubules to 37 °C in 2 mL of bath solution, two initial collections of 2–3 min at 6–8 nL/min were made. Treatments were added to the bath and tubules allowed to equilibrate for 20 min, then two further 2–3 min collections were made. To measure Pf, collected solutions were assayed for raffinose content by ultramicrofluorometry [23]; raffinose was used as a volume marker. Pf was calculated as described [16]. Biological variation in baseline osmotic water permeability between different animals has been recognized for many years [24]. Therefore, we use each tubule as its own control.

To assess the contribution of ADM to osmotic water permeability, Pf was measured in the absence of ADM, then ADM (100 nM) was added to the bath for 30 min after which 2 collections were made for raffinose determination.

To determine whether cAMP, PLC, or PKC are involved in regulating ADM-attenuated osmotic water permeability, SQ22536 (300 µM), U73122 (5 µM) and rottlerin (50 µM) were added to the bath for 20 min to inhibit cAMP, PLC, and PKC, respectively, and roflumilast (30 nM) was added to the bath for 20 min to stimulate cAMP after ADM-attenuated osmotic water permeability was measured. Then, 2 further collections were made for raffinose determination.

### 2.3. Tissue Incubation

To measure AQP2 phosphorylation, one of the two IMs from the same animal was assigned as a control; the other IM was assigned as a treatment. IMs were sectioned into ∼1 mm^3^ tissue pieces and incubated in isotonic Hanks’ balanced salt solution (HBSS). The dissecting HBSS solution contained (in mg/L): 400 KCl, 60 KH_2_PO_4_, 350 NaHCO_3_, 8000 NaCl, 90 Na_2_HPO_4_∙7H_2_O, 1000 dextrose anhydrous. The IM pieces were first incubated at 37 °C for 10 min in HBSS to establish base-line resting conditions, and then stimulated by 100 nM ADM in the treatment group. To test the effect of the inhibitors of cAMP, PLC, PKC, and cGMP, both the control and treatment groups were stimulated for 30 min with 100 nM ADM. Subsequently, SQ22536 (300 µM), U73122 (5 µM), rottlerin (50 µM), or L-NAME (5 µM) was added to the treatment group and incubation of all samples continued for 20 min. The reactions were terminated by placing the samples on ice and replacing the incubation solutions with ice-cold homogenization buffer (10 mM triethanolamine, 250 mM sucrose, 10% sodium dodecyl sulfate). Tissues were homogenized and analyzed for AQP2 phosphorylation by Western blot. AQP2 phosphorylation level was calculated as the ratio of phospho-protein/total protein to determine if the phosphorylation level per protein available changes.

### 2.4. Western Blot Analysis

Samples of whole IM protein lysate (20 µg/lane), one rat per lane, were size separated by SDS-PAGE on 12.5% gels then electroblotted to polyvinylidene difluoride (PVDF) membranes (Immobilon, Millipore, Bedford, MA, USA). Blots were blocked with 5% nonfat dry milk in Tris-buffered saline (TBS: 20 mM Tris-HCl, 0.5 M NaCl, pH 7.5) then incubated with primary antibodies overnight at 4 °C. Attached primary antibodies were identified using Alexa Fluor 680-linked anti-rabbit IgG (Molecular Probes, Eugene, OR, USA) and visualized using infrared detection with the LICOR Odyssey protein analysis system (Lincoln, NE, USA). Antibodies to AQP2 phosphorylated at serine 256 (1:2000 dilution) or 261 (1:5000 dilution) were purchased from Cell Signaling Technology, Danvers, MA, USA. Antibodies to AQP2 (1:2000 dilution) were made in our laboratory [23]. Beta tubulin (1:5000 dilution) was used to detect the loading levels of samples and purchased from Abcepta, San Diego, CA, USA. Antibodies to CRLR (1:1000 dilution) and RAMP2 (1:500 dilution) were purchased from Abcam, Cambridge, MA, USA. Antibodies to RAMP3 (1:1000 dilution) were purchased from Abcepta, San Diego, CA, USA.

### 2.5. RNA-Seq Analysis

Male Sprague–Dawley rats weighing 150–175 g, purchased from Charles River Laboratories, were used. Rats (*n* = 3) were sacrificed by decapitation. The IMs were dissected from kidney and used for RNA preparation. The total RNAs were extracted using Trizol Reagent (Invitrogen, Carlsbad, CA, USA) and purified using an RNeasy Mini Kit (Qiagen, Germantown, MD, USA). RNA sample quantity and quality were determined using a NanoDrop 2000 (Thermo Fisher Scientific, Waltham, MA, USA) and an Agilent 2100 bioanalyzer. Equal amounts of purified mRNA were transcribed to cDNA using a SMARTer PCR cDNA Synthesis Kit (Clontech, Waltham, MA, USA). Libraries were prepared according to Illumina’s instructions; cDNA was fragmented by DNase I and ligated to Illumina adapters. These adapter-ligated cDNA fragments were amplified and sequenced on the Illumina HiSeq2000 sequencer. RNA-Seq data were analyzed by an Emory core using bioinformatic software (Tophat (v 1.3.3) and cuffdiff (v 1.3.0)). The mRNA expression signals were evaluated by RPKM (reads per kilobase million). AQP2 was used as IMCD marker, AQP1 as thin descending limb (tDL) marker.

### 2.6. Measurement of cAMP Level in Inner Medulla

To measure cAMP levels, one of the two IMs from the same animal was assigned as a control; the other IM was assigned as a treatment. The IM tissues were incubated with 100 nM ADM for 30 min. The rats were sacrificed by decapitation. The IMs were removed and quickly frozen with liquid nitrogen. Then the tissues were homogenized with TCA on ice (0–4 °C) and centrifuged at 1500× *g* for 10 min. The precipitates were removed, and TCA was extracted using water-saturated ether. The supernatants were collected and assayed for cAMP level. The cAMP level of the IM tissues was determined by ELISA using an enzyme immunoassay kit from Cayman Chemical Company (Ann Arbor, MI, USA).

### 2.7. Statistics

All data are presented as means ± SE. Data from tubule perfusion studies were analyzed using a paired Student’s *t*-test [25]. Each perfused tubule serves as its own control, allowing a paired analysis of the data. Data from protein analysis studies were also analyzed using a paired Student’s *t*-test. The criterion for statistical significance is *p* < 0.05.

## 3. Results

### 3.1. ADM and Its Receptor Components (CRLR, RAMP2 and RAMP3) Are Expressed in the Inner Medulla

To determine if ADM and its receptor components are present in the IM, RNA sequencing analysis was performed on IM tissues. AQP2 was used as IMCD marker, AQP1 as tDL marker. Our results indicate that mRNAs for CRLR are detected. RAMP2 and RAMP3, but not RAMP1, are abundantly expressed in the IM. A low amount of ADM was detected in the IM (Table 1).

**Figure 1:**
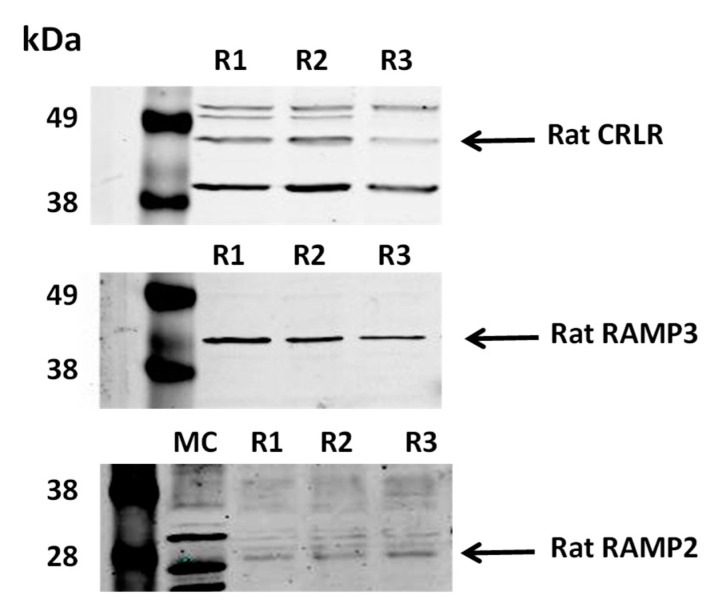
ADM receptors are expressed in rat inner medulla. Shown are Western blots of rat kidney lysates probed for CRLR, RAMP2, and RAMP3 (arrows). R1, R2, R3 are replicate rat samples. MC is a mouse control lane. Markers are in left most lanes. Lanes are separate rat lysates loaded at 30 µg/lane.

IM tissue lysates were also subject to Western analysis and probed with antibodies to CRLR, RAMP2 and RAMP3. Our results show that the 53-kDa band of CRLR protein, the 50-kDa band of RAMP3 protein, and the 20-kDa band of RAMP2 protein are detected in the IM. CRLR and RAMP3 are expressed at a significant protein level. However, RAMP2 was expressed at a very low level (Figure 1). The antibodies for CRLR, RAMP2, and RAMP3 were characterized in mice so mouse samples were used as positive controls (data not shown) to verify the Western blot results in rat tissues.

### 3.2. ADM Decreases Osmotic Water Permeability

**Figure 2:**
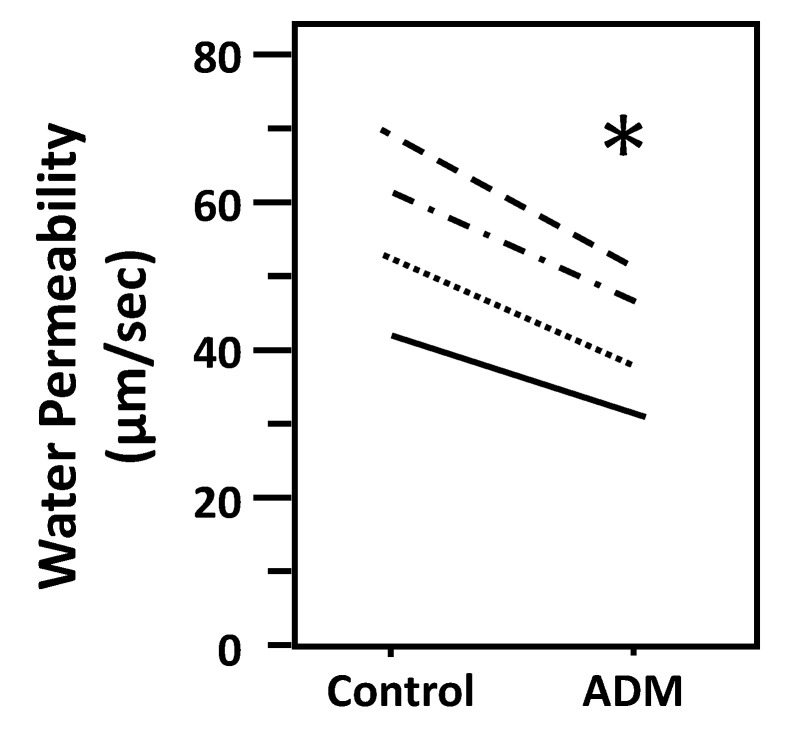
ADM decreases osmotic water permeability in rat IMCDs. Terminal IMCDs were perfused with 100 nM adrenomedullin (ADM) for 30 min. Each line represents a separate IMCD from a different rat. * *p* < 0.05 control vs. ADM, *n* = 4 rats/condition.

To determine whether ADM regulates osmotic water permeability, rat terminal IMCDs were first perfused under a basal condition without ADM and then perfused with ADM. ADM decreased Pf from 57 ± 6 to 42 ± 5 µm/s (*n* = 4, *p* < 0.05; Figure 2). To ensure that we were not observing a run-down phenomenon, we tested for time-related decreases in Pf and saw that Pf remained stable for up to 3 h (data not shown). In addition, we previously proved that 45-min incubation of tubules in the bath is enough to achieve a steady level of the basal water permeability [16].

### 3.3. ADM Phosphorylates AQP2

**Figure 3:**
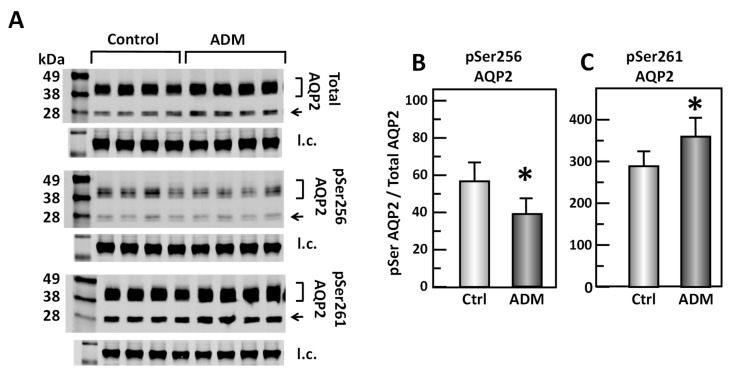
ADM decreases pSer256 AQP2 and increases pSer261 AQP2 in rat kidney. (**A**) Western analysis of kidney IM lysate from control and adrenomedullin (ADM)-treated IM probed for total (top), pSer256 (middle) and pSer 261 (bottom) AQP2. Brackets indicate the glycosylated AQP2 protein between 35 and 45 kDa and arrow indicates the un-glycosylated AQP2 protein at 29 kDa. The tubulin loading control (l.c.) is shown beneath each blot. The two kidney inner medullas from the same animal were randomly assigned into the control or the ADM groups. The matched pair comparisons were achieved by comparing Lane 1 with Lane 5, Lane 2 with Lane 6, Lane 3 with Lane 7, and Lane 4 with Lane 8. (**B**) Bar graph showing pSer256AQP2/total AQP2 density ratio. (**C**) Bar graph showing pSer261AQP2/total AQP2 density ratio. Bars: Mean ± s.e., *n* = 16, * = *p* < 0.05 control vs. ADM.

To test the role of ADM in the phosphorylation of AQP2, IM tissues were incubated in isotonic medium with ADM for 30 min. Tissue lysates were analyzed by Western blot, probing for total AQP2, pSer256 AQP2, and pSer261 AQP2 (Figure 3A). ADM significantly decreased the amount of AQP2 that was phosphorylated at serine 256 by 25% (control: 0.57 ± 0.10, ADM-treated: 0.43 ± 0.08, *n* = 16, *p* < 0.05; Figure 3B). ADM significantly increased the phosphorylation at serine 261 by 24% (control: 2.93 ± 0.35, ADM-treated: 3.62 ± 0.4, *n* = 16, *p* < 0.05; Figure 3C).

### 3.4. cAMP Regulates Osmotic Water Permeability Independently of ADM

**Figure 4:**
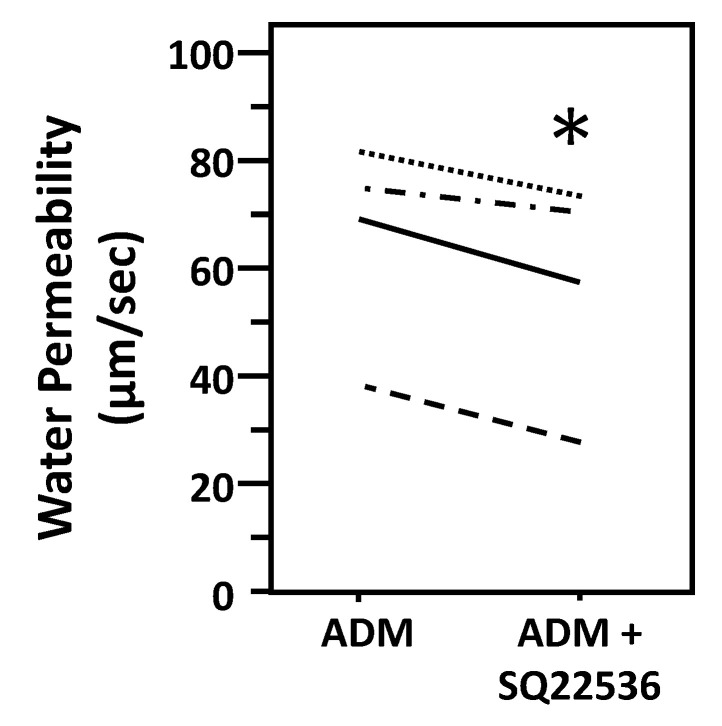
Inhibition of cAMP with SQ22536 further decreases ADM-reduced osmotic water permeability. Terminal IMCDs were perfused with 100 nM adrenomedullin (ADM) for 30 min then 300 µM SQ22536 was added and perfusion continued with ADM + SQ22536 for a further 20 min. Each line represents a separate IMCD from a different rat. * *p* < 0.05 ADM control vs. ADM + SQ22536, *n* = 4 rats/condition.

**Figure 5:**
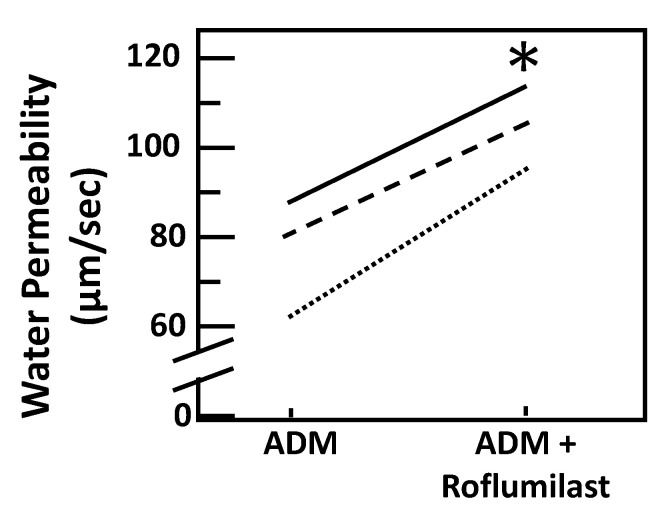
Stimulation of cAMP with the agonist roflumilast increases osmotic water permeability by ADM. Terminal IMCDs were perfused with 100 nM adrenomedullin (ADM) for 30 min then 30 nM roflumilast was added and perfusion continued with ADM + roflumilast for a further 20 min. Each line represents a separate IMCD from a different rat. * *p* < 0.05 ADM control vs. ADM + roflumilast, *n* = 3 rats/condition.

To test ADM-attenuated osmotic water permeability through a cAMP pathway, IMCDs were first perfused with ADM, then perfused with SQ22536, an adenylyl cyclase inhibitor, in the presence of ADM. SQ22536 further decreased the ADM-attenuated Pf from 66 ± 10 to 58 ± 11 µm/s (*n* = 4, *p* < 0.05; Figure 4). To further identify the role of cAMP in ADM-attenuated osmotic water permeability, IMCDs were perfused with ADM, then perfused with roflumilast, a phosphodiesterase inhibitor, in the presence of ADM. Roflumilast increased the ADM-attenuated Pf from 73.42 ± 10.68 to 103.83 ± 5.48 µm/s (*n* = 3, *p* < 0.05; Figure 5).

### 3.5. ADM Increases cAMP Level

**Figure 6:**
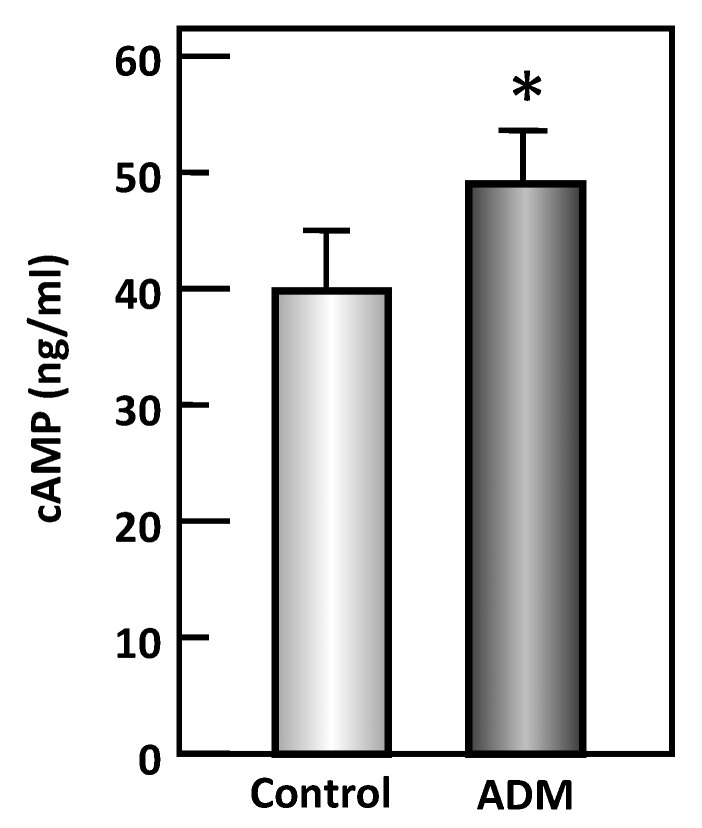
ADM increases the cAMP level in rat inner medulla. Rat kidney inner medullas were treated with (1 kidney IM) or without (contralateral kidney IM) 100 nM adrenomedullin (ADM), then lysates prepared and analyzed by ELISA. Bars: mean ± s.e., *n* = kidneys from 7 individual rats, * = *p* < 0.05 control vs. ADM.

To determine whether ADM stimulates cAMP production, IM tissue lysates were analyzed for cAMP level in response to ADM. ADM significantly increased cAMP level by 20% (control: 40.68 ± 5.20 ng/mL, ADM treated: 48.78 ± 4.50 ng/mL, *n* = 7, *p* < 0.05; Figure 6).

### 3.6. Inhibition of PLC Increases Osmotic Water Permeability

**Figure 7:**
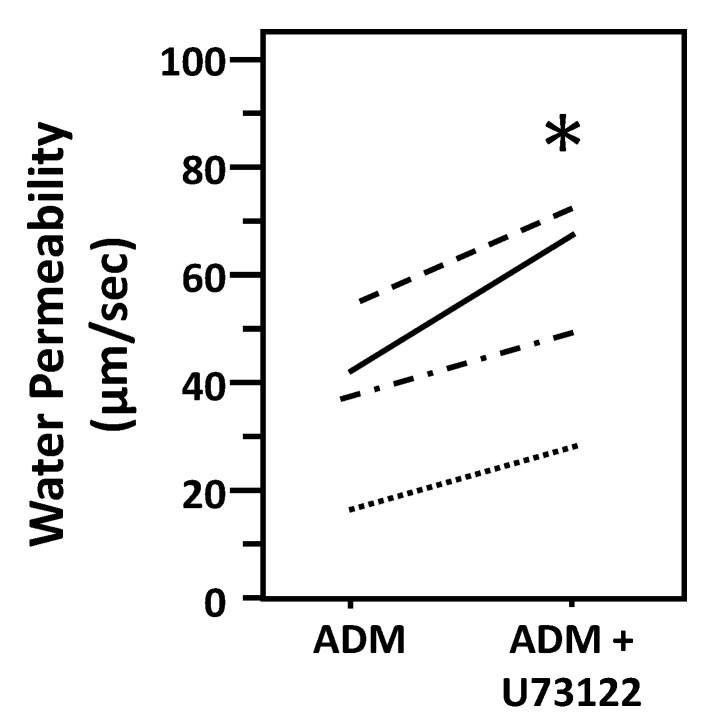
Inhibition of Phospholipase C by U73122 reverses the decrease in osmotic water permeability by ADM. Terminal IMCDs were perfused with 100 nM adrenomedullin (ADM) for 30 min then 10 µM U73122 was added and perfusion continued with ADM+U73122 for a further 20 min. Each line represents a separate IMCD from a different rat. * *p* < 0.05 ADM control vs. ADM + U73122, *n* = 4 rats/condition.

To test whether ADM-attenuated osmotic water permeability through a PLC pathway, IMCDs were first perfused with ADM, then perfused with U73122, a PLC inhibitor, in the presence of ADM. U73122 increased Pf from 37 ± 8 to 54 ± 10 µm/s (*n* = 4, *p* < 0.05; Figure 7).

### 3.7. Inhibition of PLC Changes ADM-Mediated AQP2 Phosphorylation

**Figure 8:**
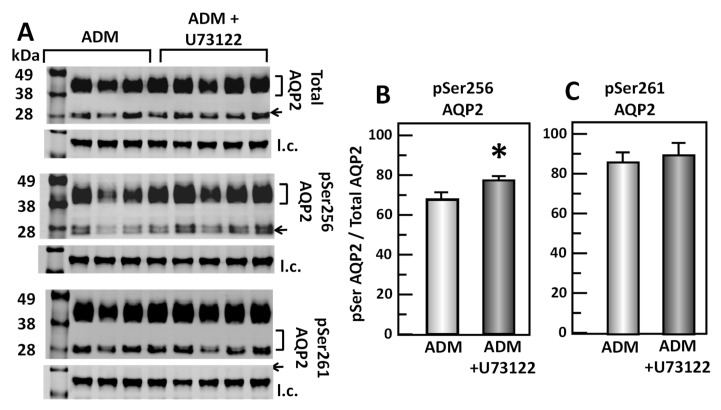
Inhibition of Phospholipase C by U73122 significantly increases pSer256 at AQP2 but does not change pSer261. (**A**) Western analysis of kidney IM lysate from ADM control and ADM plus U73122-treated IM probed for total (top), pSer256 (middle), and pSer 261 (bottom) AQP2. Brackets indicate the glycosylated AQP2 protein between 35 and 45 kDa and arrow indicates the un-glycosylated AQP2 protein at 29 kDa. The tubulin loading control (l.c.) is shown beneath each blot. The two kidney inner medullas from the same animal were randomly assigned into the ADM control or the ADM+U73122 groups. The matched pair comparisons were achieved by comparing Lane 1 with Lane 5, Lane 2 with Lane 6, Lane 3 with Lane 7, and Lane 4 with Lane 8. (**B**) Bar graph showing pSer256AQP2/total AQP2 density ratio. (**C**) Bar graph showing pSer261AQP2/total AQP2 density ratio. Bars: Mean ± s.e., *n* = 8, * = *p* < 0.05 ADM control vs. ADM + U73122.

To test the role of U73122, a PLC inhibitor, in the ADM-mediated phosphorylation of AQP2, IM tissues were incubated in isotonic medium with ADM for 30 min and then treated with U73122 for 20 min. Tissue lysates were analyzed by Western blot, probing for total AQP2, pSer256 AQP2, and pSer261 AQP2 (Figure 8A). Inhibition of PLC by U73122 significantly increased pSer256 AQP2 by 15% (ADM control: 0.67 ± 0.03, U73122-treated: 0.77 ± 0.03, *n* = 8, *p* < 0.05; Figure 8B). However, inhibition of PLC by U73122 did not significantly change the pSer261 AQP2 (ADM control: 0.86 ± 0.06, U73122-treated: 0.89 ± 0.06, *n* = 8; Figure 8C).

### 3.8. Inhibition of PKC Increases Osmotic Water Permeability

**Figure 9:**
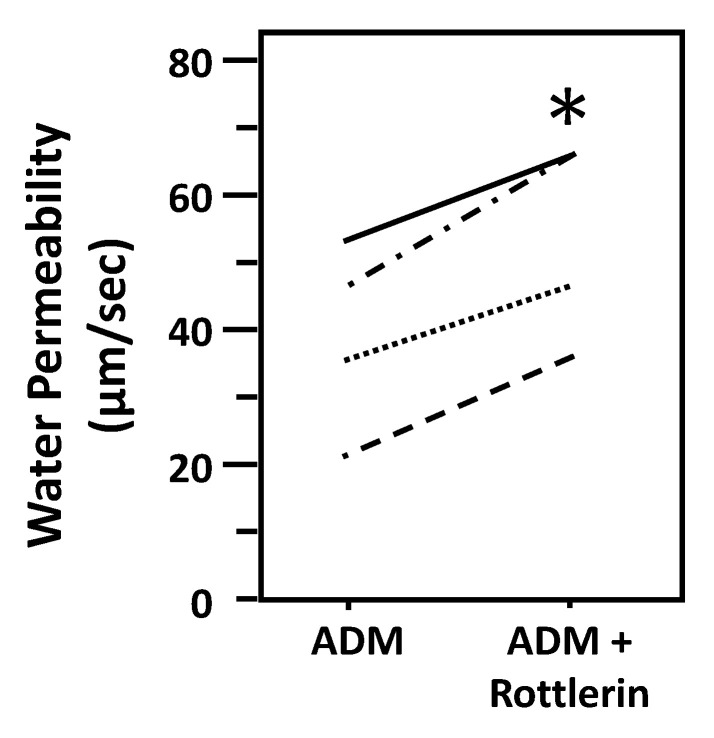
Inhibition of protein kinase C by rottlerin reverses the decrease in osmotic water permeability by ADM. Terminal IMCDs were perfused with 100 nM adrenomedullin (ADM) for 30 min then 50 µM rottlerin was added and perfusion continued with ADM + rottlerin for a further 20 min. Each line represents a separate IMCD from a different rat. * *p* < 0.05 ADM control vs. ADM + rottlerin, *n* = 4 rats/condition.

To test whether ADM-attenuated osmotic water permeability through a PKC pathway, IMCDs were first perfused with ADM, then perfused with rottlerin, a PKC inhibitor, in the presence of ADM. Rottlerin increased Pf from 38 ± 7 to 52 ± 6 µm/s (*n* = 4, *p* < 0.05; Figure 9).

### 3.9. Inhibition of PKC Alters ADM-Mediated AQP2 Phosphorylation

**Figure 10:**
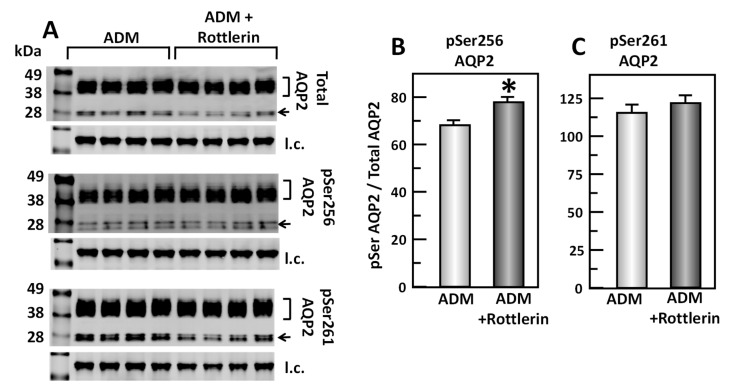
Inhibition of Protein Kinase C by rottlerin significantly increases pSer256 at AQP2 but does not change pSer261. (**A**) Western analysis of kidney IM lysate from ADM control and ADM plus rottlerin-treated IM probed for total (top), pSer256 (middle), and pSer 261 (bottom) AQP2. Brackets indicate the glycosylated AQP2 protein between 35 and 45 kDa and arrow indicates the un-glycosylated AQP2 protein at 29 kDa. The tubulin loading control (l.c.) is shown beneath each blot. The two kidney inner medullas from the same animal were randomly assigned into the ADM control or the ADM + rottlerin groups. The matched pair comparisons were achieved by comparing Lane 1 with Lane 5, Lane 2 with Lane 6, Lane 3 with Lane 7, and Lane 4 with Lane 8. (**B**) Bar graph showing pSer256AQP2/total AQP2 density ratio. (**C**) Bar graph showing pSer261AQP2/total AQP2 density ratio. Bars: Mean ± s.e., *n* = 4, * = *p* < 0.05 ADM control vs. ADM + rottlerin.

To test the effect of rottlerin, a PKC inhibitor, in the ADM-mediated phosphorylation of AQP2, IM tissues were incubated in isotonic medium with ADM for 30 min and then treated with rottlerin for 20 min. Tissue lysates were analyzed by Western blot, probing for total AQP2, pSer256 AQP2, and pSer261 AQP2 (Figure 10A). Inhibition of PKC by rottlerin significantly increased pSer256 AQP2 by 11% (ADM control: 0.68 ± 0.02, rottlerin-treated: 0.75 ± 0.01, *n* = 4, *p* < 0.05; Figure 10B). However, inhibition of PKC by rottlerin did not significantly change the pSer261 AQP2 (ADM control: 1.15 ± 0.05, rottlerin-treated: 1.21 ± 0.04, *n* = 4; Figure 10C).

### 3.10. Inhibition of cGMP Does Not Change ADM-Mediated AQP2 Phosphorylation

**Figure 11:**
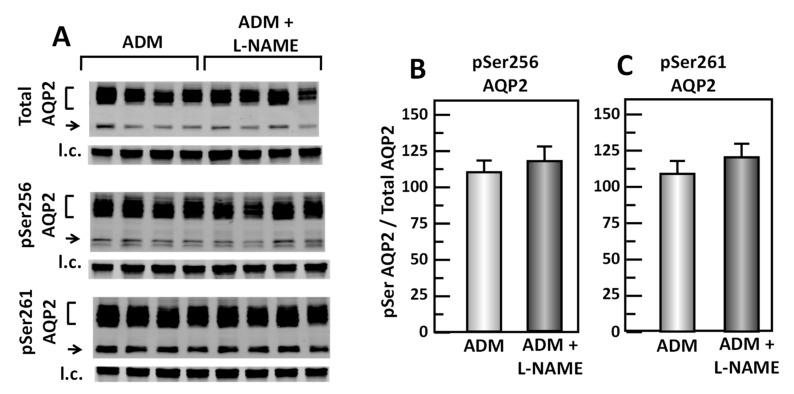
Inhibition of cGMP by L-NAME does not change pSer256 and pSer261 at AQP2. (**A**) Western analysis of kidney IM lysate from ADM control and ADM plus L-NAME-treated IM probed for total (top), pSer256 (middle), and pSer 261 (bottom) AQP2. Brackets indicate the glycosylated AQP2 protein between 35 and 45 kDa and arrow indicates the un-glycosylated AQP2 protein at 29 kDa. The tubulin loading control (l.c.) is shown beneath each blot. The two kidney inner medullas from the same animal were randomly assigned to the ADM control or the ADM+L-NAME groups. The matched pair comparisons were achieved by comparing Lane 1 with Lane 5, Lane 2 with Lane 6, Lane 3 with Lane 7, and Lane 4 with Lane 8. (**B**) Bar graph showing pSer256AQP2/total AQP2 density ratio. (**C**) Bar graph showing pSer261AQP2/total AQP2 density ratio. Bars: Mean ± s.e., *n* = 4.

To test whether cGMP regulates the ADM-mediated phosphorylation of AQP2, IM tissues were incubated in isotonic medium with ADM for 30 min and then treated with L-NAME for 20 min. Tissue lysates were analyzed by western blot, probing for total AQP2, pSer256 AQP2, and pSer261 AQP2 (Figure 11A). Inhibition of cGMP by L-NAME did not change pSer256 AQP2 (ADM control: 1.11 ± 0.08, L-NAME-treated: 1.19 ± 0.09, *n* = 4; Figure 11B) and the pSer261 AQP2 (ADM control: 1.10 ± 0.08, L-NAME-treated: 1.21 ± 0.07, *n* = 4; Figure 11C).

## 4. Discussion

Water reabsorption is mainly regulated by vasopressin through cAMP-dependent signaling pathways [16,26,27,28]. Recent studies demonstrate that local, paracrine, and autocrine hormones such as ADM are also involved in the regulation of fluid and electrolyte homeostasis [5,12]. ADM is expressed in the kidney [8,10]. However, limited studies have been done to elucidate the renal effect of ADM on water reabsorption. This study provides evidence that ADM decreases osmotic water permeability through PLC-PKC signaling pathways.

Previous studies show that ADM and ADM receptors are detected in the kidney [6,9]. But the expression of ADM receptors is not well defined. Analysis of mRNA showed that ADM mRNA is most abundant in the cortex. In contrast, mRNA for ADM receptors is most abundant in the IM [11]. But that study did not address which specific ADM receptor type, CRLR/RAMP2 or CRLR/RAMP3, is expressed in the kidney. Immunohistochemistry analysis showed that RAMP3 is mainly expressed in collecting duct cells, while RAMP2 is not expressed on tubular cells in normal kidney tissues [29]. However, the expression of CRLR was not tested in this immunohistochemistry analysis. To better understand the distribution of ADM receptors in rat IM, we analyzed the mRNAs for ADM, CRLR, RAMP2, and RAMP3. Our results show that mRNAs for ADM, CRLR, RAMP2, and RAMP3 are present in IM (Table 1). A low amount of ADM is detected, which suggests ADM exerts an autocrine and/or paracrine regulation mechanism in the IM. Our findings for the location of mRNA of ADM and its receptors are consistent with those reported by Knepper’s group [30]. The small difference could come from different materials or dissected tubules vs. IM tissues. IM tissues contain IMCDs but also small amounts of interstitial tissues including capillary vessels. In addition, IM also contains a small portion of long limbs of Henle loop. We compared these two data sets: a low (marginal) amount of ADM was identified in both IMCD (Knepper’s data) and IM (our data). We saw a low level of CRLR in IM but this was not seen by the Knepper group in IMCD. Neither we nor the Knepper group saw any RAMP1 in either IMCD or IM. We both observed RAMP2 (in IMCD and IM). RAMP3 is reported to be in a thin ascending limb by the Knepper group and we were able to see both RAMP3 mRNA and protein in IM.

We also performed Western blot using specific antibodies raised against CRLR, RAMP2 and RAMP3. Our results show a significant protein expression of CRLR and RAMP3. In contrast, RAMP2 is expressed at a very low level (Figure 1), which is consistent with the finding that RAMP2 protein is not expressed on tubular cells in the immunohistochemistry analysis [29]. Our results suggest that CRLR-RAMP3 is the predominant receptor that ADM interacts with rat IM. Although the mRNA for RAMP2 is present in rat IM, the translation of mRNA to protein may be blocked by unknown mechanisms. It is worthwhile to mention that the molecular weight of CRLR varies among various species depending on the level of glycosylation [31]. RAMP3 is also present in three different types: RAMP3 monomer (28 kDa), RAMP3 homodimer (50 kDa), and RAMP3 heterodimer (73–75 kDa) [32]. Our results indicate that the molecular weight of CRLR is 53 kDa and RAMP3 is a homodimer with a molecular weight of 50 kDa in rat IM.

Initially, ADM was identified as a cAMP stimulator via activation of adenylyl cyclase [1,2,6]. cAMP is involved in vasopressin-stimulated water reabsorption in the kidney [16,26,27,28]. Given its ability to activate cAMP, we expected that ADM may also stimulate water reabsorption through cAMP. Therefore, we tested the osmotic water permeability in isolated perfused IMCDs. Surprisingly, ADM decreases osmotic water permeability in IMCDs (Figure 2) despite an increase in cAMP level (Figure 6), which supports the finding that intrarenal arterial infusion of ADM causes diuresis, not anti-diuresis [13,14]. ADM decreases the phosphorylation of AQP2 at serine 256, whereas it increases phosphorylation of AQP2 at serine 261 (Figure 3). Phosphorylation of AQP2 at serine 256 promotes AQP2 trafficking to the apical plasma membrane [33], and phosphorylation of AQP2 at serine 261 is linked to ubiquitination [34]. This suggests that ADM decreases plasma membrane accumulation of AQP2 through phosphorylation at serine 256 and increases ubiquitination of AQP2 associated with phosphorylation at serine 261. The data in the present study demonstrated that ADM regulates osmotic water permeability independent of cAMP pathways even though ADM increases the cAMP level. To verify the role of cAMP in ADM-regulated water permeability, we tested whether an adenylyl cyclase inhibitor, SQ22536, affects ADM-attenuated water permeability. Our results show that inhibition of cAMP by SQ22536 further decreased ADM-attenuated water permeability (Figure 4). We also tested whether a phosphodiesterase inhibitor, roflumilast, affects ADM-attenuated water permeability. PDE4b specifically catalyzes cAMP hydrolysis. Roflumilast is a blocker of PDE4, which could lead to increased cAMP. Our results show that stimulation of cAMP by roflumilast increased ADM-attenuated water permeability (Figure 5). Obviously, cAMP regulates water reabsorption in its own way and does not interfere with ADM-regulated water permeability. To confirm that the nonintervention of cAMP in ADM-attenuated water permeability is not due to the deficiency of cAMP, we tested cAMP production in the IM. Our result indicated that ADM significantly increases cAMP level (Figure 6). However, we admit that we cannot tell IMCDs from thin limbs in the cAMP measurements in IM. Our result suggested that ADM increases the cAMP level in either IMCDs, or thin limbs, or both. Previous studies [6,11] showed that the cAMP level is increased in IMCDs in response to ADM. Therefore, it is likely that the ADM-stimulated cAMP level in this study results primarily, but not exclusively, from IMCDs.

The inconsistency of the role of cAMP in regulation of osmotic water permeability may be explained by the concept of compartmentalized cAMP signaling [35,36]. cAMP signaling pathways control a variety of cellular processes in a stimulation-specific manner [36]. ADM may stimulate a different cAMP pool from that stimulated by vasopressin. Thus, ADM-stimulated cAMP may not be able to activate PKA and further increase water reabsorption. The alternative explanation is that other signaling pathways stimulated by ADM have a robust inhibitory influence and counteract the stimulatory effect of cAMP on osmotic water permeability.

In addition to cAMP, ADM stimulates PLC through G protein-coupled receptors [5]. PLC further activates PKC and cGMP [5]. Previous studies indicated that ADM regulates some cellular functions in cAMP-independent pathways. ADM enhances cardiac contractility via cAMP-independent mechanisms including activation of PKC [37]. ADM induced vasorelaxation in the rat aorta and kidney via a NO-cGMP pathway [38]. To determine whether the decrease in osmotic water permeability by ADM is in response to the activation of PLC, the PLC inhibitor, U73122, was used to reverse the decreased osmotic water permeability. Data from isolated perfused tubules show that U73122 significantly increases ADM-attenuated water permeability (Figure 7), which supports the concept that ADM decreases water permeability by stimulating cAMP-independent pathways. U73122 significantly increased phosphorylation at serine 256 but did not change phosphorylation of AQP2 at serine 261 (Figure 8), which suggests that the increase in ADM-attenuated water permeability by PLC inhibition may be related to the increase in AQP2 trafficking to the plasma membrane instead of a decrease in endocytosis of AQP2.

Given that both PKC and cGMP are downstream signals of PLC signaling pathways, it is likely that PKC and/or cGMP are involved in ADM-regulated water reabsorption. To investigate whether PKC regulates ADM-attenuated water permeability, the PKC inhibitor, rottlerin, was used to reverse the osmotic water permeability. Our data show that inhibition of PKC by rottlerin significantly increases ADM-attenuated water permeability (Figure 9), which indicates that ADM regulates water transport through PKC. Blocking PKC with rottlerin increased phosphorylation of AQP2 at serine 256 but did not alter phosphorylation of AQP2 at serine 261 (Figure 10), which is consistent with the effect of inhibition of PLC with U73122. To determine whether cGMP changes ADM-mediated phosphorylation of AQP2, the nitric oxide synthase inhibitor, L-NAME was used to block the phosphorylation of AQP2 by ADM. Our results show that inhibition of cGMP does not change AQP2 phosphorylation at serine 256 and serine 261 (Figure 11), which suggests that ADM does not regulate water permeability through cGMP. Therefore, we verified that ADM reduces osmotic water permeability through a PLC-PKC signaling pathway. However, we need to point out that ADM may also involve other signaling pathways to regulate water reabsorption because the PLC-PKC signaling pathway does not change phosphorylation of AQP2 at serine 261. How ADM increases phosphorylation of AQP2 at serine 261 needs to be further investigated.

In conclusion, the present study shows that ADM and its receptors are expressed in rat inner medulla. ADM stimulates cAMP production, but ADM regulates water reabsorption through cAMP-independent pathways. ADM reduces water transport by activating the PLC-PKC signaling pathway. The decrease in water permeability appears to be a result of a decrease in AQP2 phosphorylation at serine 256, which may impede AQP2 trafficking to the plasma membrane. ADM is a potential therapeutic target for treatment of cardiovascular diseases associated with water imbalance.

## Figures and Tables

**Table 1:** mRNA expression for ADM and its receptor components in rat inner medulla.

Gene ID	mRNA Expression (RPKM, *n* = 3)
ADM	0.48 ± 0.13
CRLR	3.51 ± 2.17
RAMP1	No Expression
RAMP2	21.09 ± 7.01
RAMP3	17.62 ± 2.62
AQP1 (tDL marker)	2.59 ± 0.58
AQP2 (IMCD marker)	130.8 ± 51.1
